# 77 A Comparison of Large Tbsa Scald Versus Flame Burns in Young Children

**DOI:** 10.1093/jbcr/irad045.051

**Published:** 2023-05-15

**Authors:** Ashley Daniel, Jenna Miller, Emily Brier, Jennifer Flint, Marita Thompson, Pablo Aguayo, Kelli Patterson, Daniel Marx, Grace Mallampalli, Justin Klein, Lisa Vitale, Mariah Malaniak, Christina Shanti, Erica Hodgman, Rebecca Gardner, Alejandro Garcia

**Affiliations:** Children's Mercy Hospital, Kansas City, Missouri; Children’s Mercy Kansas City, Kansas City, Missouri; Children’s Mercy Hospital, Lees Summit, Missouri; Children's Mercy Hospital, Kansas City, Missouri; Dell Children's Medical Center, Austin, Texas; Children's Mercy Kansas City, Kansas City, Missouri; Ohio State University; Nationwide Children's Hospital, Columbus, Ohio; Children's Mercy Kansas City, Kansas City, Missouri; The Ohio State University College of Medicine; Nationwide Children's Hospital, Columbus, Ohio; Children's Hospital of Michigan, Detroit, Michigan; Children's Hospital of Michigan, Detroit, Michigan; Children's Hospital of Michigan, Flat Rock, Michigan; Department of Surgery, Division of Pediatric Surgery, Children's Hospital of Michigan/Wayne State University School of Medicine, Detroit, Michigan; John’s Hopkins Children’s Center, Baltimore, Maryland; Johns Hopkins Hospital, Baltimore, Maryland; Johns Hopkins Medicine, Baltimore, Maryland; Children's Mercy Hospital, Kansas City, Missouri; Children’s Mercy Kansas City, Kansas City, Missouri; Children’s Mercy Hospital, Lees Summit, Missouri; Children's Mercy Hospital, Kansas City, Missouri; Dell Children's Medical Center, Austin, Texas; Children's Mercy Kansas City, Kansas City, Missouri; Ohio State University; Nationwide Children's Hospital, Columbus, Ohio; Children's Mercy Kansas City, Kansas City, Missouri; The Ohio State University College of Medicine; Nationwide Children's Hospital, Columbus, Ohio; Children's Hospital of Michigan, Detroit, Michigan; Children's Hospital of Michigan, Detroit, Michigan; Children's Hospital of Michigan, Flat Rock, Michigan; Department of Surgery, Division of Pediatric Surgery, Children's Hospital of Michigan/Wayne State University School of Medicine, Detroit, Michigan; John’s Hopkins Children’s Center, Baltimore, Maryland; Johns Hopkins Hospital, Baltimore, Maryland; Johns Hopkins Medicine, Baltimore, Maryland; Children's Mercy Hospital, Kansas City, Missouri; Children’s Mercy Kansas City, Kansas City, Missouri; Children’s Mercy Hospital, Lees Summit, Missouri; Children's Mercy Hospital, Kansas City, Missouri; Dell Children's Medical Center, Austin, Texas; Children's Mercy Kansas City, Kansas City, Missouri; Ohio State University; Nationwide Children's Hospital, Columbus, Ohio; Children's Mercy Kansas City, Kansas City, Missouri; The Ohio State University College of Medicine; Nationwide Children's Hospital, Columbus, Ohio; Children's Hospital of Michigan, Detroit, Michigan; Children's Hospital of Michigan, Detroit, Michigan; Children's Hospital of Michigan, Flat Rock, Michigan; Department of Surgery, Division of Pediatric Surgery, Children's Hospital of Michigan/Wayne State University School of Medicine, Detroit, Michigan; John’s Hopkins Children’s Center, Baltimore, Maryland; Johns Hopkins Hospital, Baltimore, Maryland; Johns Hopkins Medicine, Baltimore, Maryland; Children's Mercy Hospital, Kansas City, Missouri; Children’s Mercy Kansas City, Kansas City, Missouri; Children’s Mercy Hospital, Lees Summit, Missouri; Children's Mercy Hospital, Kansas City, Missouri; Dell Children's Medical Center, Austin, Texas; Children's Mercy Kansas City, Kansas City, Missouri; Ohio State University; Nationwide Children's Hospital, Columbus, Ohio; Children's Mercy Kansas City, Kansas City, Missouri; The Ohio State University College of Medicine; Nationwide Children's Hospital, Columbus, Ohio; Children's Hospital of Michigan, Detroit, Michigan; Children's Hospital of Michigan, Detroit, Michigan; Children's Hospital of Michigan, Flat Rock, Michigan; Department of Surgery, Division of Pediatric Surgery, Children's Hospital of Michigan/Wayne State University School of Medicine, Detroit, Michigan; John’s Hopkins Children’s Center, Baltimore, Maryland; Johns Hopkins Hospital, Baltimore, Maryland; Johns Hopkins Medicine, Baltimore, Maryland; Children's Mercy Hospital, Kansas City, Missouri; Children’s Mercy Kansas City, Kansas City, Missouri; Children’s Mercy Hospital, Lees Summit, Missouri; Children's Mercy Hospital, Kansas City, Missouri; Dell Children's Medical Center, Austin, Texas; Children's Mercy Kansas City, Kansas City, Missouri; Ohio State University; Nationwide Children's Hospital, Columbus, Ohio; Children's Mercy Kansas City, Kansas City, Missouri; The Ohio State University College of Medicine; Nationwide Children's Hospital, Columbus, Ohio; Children's Hospital of Michigan, Detroit, Michigan; Children's Hospital of Michigan, Detroit, Michigan; Children's Hospital of Michigan, Flat Rock, Michigan; Department of Surgery, Division of Pediatric Surgery, Children's Hospital of Michigan/Wayne State University School of Medicine, Detroit, Michigan; John’s Hopkins Children’s Center, Baltimore, Maryland; Johns Hopkins Hospital, Baltimore, Maryland; Johns Hopkins Medicine, Baltimore, Maryland; Children's Mercy Hospital, Kansas City, Missouri; Children’s Mercy Kansas City, Kansas City, Missouri; Children’s Mercy Hospital, Lees Summit, Missouri; Children's Mercy Hospital, Kansas City, Missouri; Dell Children's Medical Center, Austin, Texas; Children's Mercy Kansas City, Kansas City, Missouri; Ohio State University; Nationwide Children's Hospital, Columbus, Ohio; Children's Mercy Kansas City, Kansas City, Missouri; The Ohio State University College of Medicine; Nationwide Children's Hospital, Columbus, Ohio; Children's Hospital of Michigan, Detroit, Michigan; Children's Hospital of Michigan, Detroit, Michigan; Children's Hospital of Michigan, Flat Rock, Michigan; Department of Surgery, Division of Pediatric Surgery, Children's Hospital of Michigan/Wayne State University School of Medicine, Detroit, Michigan; John’s Hopkins Children’s Center, Baltimore, Maryland; Johns Hopkins Hospital, Baltimore, Maryland; Johns Hopkins Medicine, Baltimore, Maryland; Children's Mercy Hospital, Kansas City, Missouri; Children’s Mercy Kansas City, Kansas City, Missouri; Children’s Mercy Hospital, Lees Summit, Missouri; Children's Mercy Hospital, Kansas City, Missouri; Dell Children's Medical Center, Austin, Texas; Children's Mercy Kansas City, Kansas City, Missouri; Ohio State University; Nationwide Children's Hospital, Columbus, Ohio; Children's Mercy Kansas City, Kansas City, Missouri; The Ohio State University College of Medicine; Nationwide Children's Hospital, Columbus, Ohio; Children's Hospital of Michigan, Detroit, Michigan; Children's Hospital of Michigan, Detroit, Michigan; Children's Hospital of Michigan, Flat Rock, Michigan; Department of Surgery, Division of Pediatric Surgery, Children's Hospital of Michigan/Wayne State University School of Medicine, Detroit, Michigan; John’s Hopkins Children’s Center, Baltimore, Maryland; Johns Hopkins Hospital, Baltimore, Maryland; Johns Hopkins Medicine, Baltimore, Maryland; Children's Mercy Hospital, Kansas City, Missouri; Children’s Mercy Kansas City, Kansas City, Missouri; Children’s Mercy Hospital, Lees Summit, Missouri; Children's Mercy Hospital, Kansas City, Missouri; Dell Children's Medical Center, Austin, Texas; Children's Mercy Kansas City, Kansas City, Missouri; Ohio State University; Nationwide Children's Hospital, Columbus, Ohio; Children's Mercy Kansas City, Kansas City, Missouri; The Ohio State University College of Medicine; Nationwide Children's Hospital, Columbus, Ohio; Children's Hospital of Michigan, Detroit, Michigan; Children's Hospital of Michigan, Detroit, Michigan; Children's Hospital of Michigan, Flat Rock, Michigan; Department of Surgery, Division of Pediatric Surgery, Children's Hospital of Michigan/Wayne State University School of Medicine, Detroit, Michigan; John’s Hopkins Children’s Center, Baltimore, Maryland; Johns Hopkins Hospital, Baltimore, Maryland; Johns Hopkins Medicine, Baltimore, Maryland; Children's Mercy Hospital, Kansas City, Missouri; Children’s Mercy Kansas City, Kansas City, Missouri; Children’s Mercy Hospital, Lees Summit, Missouri; Children's Mercy Hospital, Kansas City, Missouri; Dell Children's Medical Center, Austin, Texas; Children's Mercy Kansas City, Kansas City, Missouri; Ohio State University; Nationwide Children's Hospital, Columbus, Ohio; Children's Mercy Kansas City, Kansas City, Missouri; The Ohio State University College of Medicine; Nationwide Children's Hospital, Columbus, Ohio; Children's Hospital of Michigan, Detroit, Michigan; Children's Hospital of Michigan, Detroit, Michigan; Children's Hospital of Michigan, Flat Rock, Michigan; Department of Surgery, Division of Pediatric Surgery, Children's Hospital of Michigan/Wayne State University School of Medicine, Detroit, Michigan; John’s Hopkins Children’s Center, Baltimore, Maryland; Johns Hopkins Hospital, Baltimore, Maryland; Johns Hopkins Medicine, Baltimore, Maryland; Children's Mercy Hospital, Kansas City, Missouri; Children’s Mercy Kansas City, Kansas City, Missouri; Children’s Mercy Hospital, Lees Summit, Missouri; Children's Mercy Hospital, Kansas City, Missouri; Dell Children's Medical Center, Austin, Texas; Children's Mercy Kansas City, Kansas City, Missouri; Ohio State University; Nationwide Children's Hospital, Columbus, Ohio; Children's Mercy Kansas City, Kansas City, Missouri; The Ohio State University College of Medicine; Nationwide Children's Hospital, Columbus, Ohio; Children's Hospital of Michigan, Detroit, Michigan; Children's Hospital of Michigan, Detroit, Michigan; Children's Hospital of Michigan, Flat Rock, Michigan; Department of Surgery, Division of Pediatric Surgery, Children's Hospital of Michigan/Wayne State University School of Medicine, Detroit, Michigan; John’s Hopkins Children’s Center, Baltimore, Maryland; Johns Hopkins Hospital, Baltimore, Maryland; Johns Hopkins Medicine, Baltimore, Maryland; Children's Mercy Hospital, Kansas City, Missouri; Children’s Mercy Kansas City, Kansas City, Missouri; Children’s Mercy Hospital, Lees Summit, Missouri; Children's Mercy Hospital, Kansas City, Missouri; Dell Children's Medical Center, Austin, Texas; Children's Mercy Kansas City, Kansas City, Missouri; Ohio State University; Nationwide Children's Hospital, Columbus, Ohio; Children's Mercy Kansas City, Kansas City, Missouri; The Ohio State University College of Medicine; Nationwide Children's Hospital, Columbus, Ohio; Children's Hospital of Michigan, Detroit, Michigan; Children's Hospital of Michigan, Detroit, Michigan; Children's Hospital of Michigan, Flat Rock, Michigan; Department of Surgery, Division of Pediatric Surgery, Children's Hospital of Michigan/Wayne State University School of Medicine, Detroit, Michigan; John’s Hopkins Children’s Center, Baltimore, Maryland; Johns Hopkins Hospital, Baltimore, Maryland; Johns Hopkins Medicine, Baltimore, Maryland; Children's Mercy Hospital, Kansas City, Missouri; Children’s Mercy Kansas City, Kansas City, Missouri; Children’s Mercy Hospital, Lees Summit, Missouri; Children's Mercy Hospital, Kansas City, Missouri; Dell Children's Medical Center, Austin, Texas; Children's Mercy Kansas City, Kansas City, Missouri; Ohio State University; Nationwide Children's Hospital, Columbus, Ohio; Children's Mercy Kansas City, Kansas City, Missouri; The Ohio State University College of Medicine; Nationwide Children's Hospital, Columbus, Ohio; Children's Hospital of Michigan, Detroit, Michigan; Children's Hospital of Michigan, Detroit, Michigan; Children's Hospital of Michigan, Flat Rock, Michigan; Department of Surgery, Division of Pediatric Surgery, Children's Hospital of Michigan/Wayne State University School of Medicine, Detroit, Michigan; John’s Hopkins Children’s Center, Baltimore, Maryland; Johns Hopkins Hospital, Baltimore, Maryland; Johns Hopkins Medicine, Baltimore, Maryland; Children's Mercy Hospital, Kansas City, Missouri; Children’s Mercy Kansas City, Kansas City, Missouri; Children’s Mercy Hospital, Lees Summit, Missouri; Children's Mercy Hospital, Kansas City, Missouri; Dell Children's Medical Center, Austin, Texas; Children's Mercy Kansas City, Kansas City, Missouri; Ohio State University; Nationwide Children's Hospital, Columbus, Ohio; Children's Mercy Kansas City, Kansas City, Missouri; The Ohio State University College of Medicine; Nationwide Children's Hospital, Columbus, Ohio; Children's Hospital of Michigan, Detroit, Michigan; Children's Hospital of Michigan, Detroit, Michigan; Children's Hospital of Michigan, Flat Rock, Michigan; Department of Surgery, Division of Pediatric Surgery, Children's Hospital of Michigan/Wayne State University School of Medicine, Detroit, Michigan; John’s Hopkins Children’s Center, Baltimore, Maryland; Johns Hopkins Hospital, Baltimore, Maryland; Johns Hopkins Medicine, Baltimore, Maryland; Children's Mercy Hospital, Kansas City, Missouri; Children’s Mercy Kansas City, Kansas City, Missouri; Children’s Mercy Hospital, Lees Summit, Missouri; Children's Mercy Hospital, Kansas City, Missouri; Dell Children's Medical Center, Austin, Texas; Children's Mercy Kansas City, Kansas City, Missouri; Ohio State University; Nationwide Children's Hospital, Columbus, Ohio; Children's Mercy Kansas City, Kansas City, Missouri; The Ohio State University College of Medicine; Nationwide Children's Hospital, Columbus, Ohio; Children's Hospital of Michigan, Detroit, Michigan; Children's Hospital of Michigan, Detroit, Michigan; Children's Hospital of Michigan, Flat Rock, Michigan; Department of Surgery, Division of Pediatric Surgery, Children's Hospital of Michigan/Wayne State University School of Medicine, Detroit, Michigan; John’s Hopkins Children’s Center, Baltimore, Maryland; Johns Hopkins Hospital, Baltimore, Maryland; Johns Hopkins Medicine, Baltimore, Maryland; Children's Mercy Hospital, Kansas City, Missouri; Children’s Mercy Kansas City, Kansas City, Missouri; Children’s Mercy Hospital, Lees Summit, Missouri; Children's Mercy Hospital, Kansas City, Missouri; Dell Children's Medical Center, Austin, Texas; Children's Mercy Kansas City, Kansas City, Missouri; Ohio State University; Nationwide Children's Hospital, Columbus, Ohio; Children's Mercy Kansas City, Kansas City, Missouri; The Ohio State University College of Medicine; Nationwide Children's Hospital, Columbus, Ohio; Children's Hospital of Michigan, Detroit, Michigan; Children's Hospital of Michigan, Detroit, Michigan; Children's Hospital of Michigan, Flat Rock, Michigan; Department of Surgery, Division of Pediatric Surgery, Children's Hospital of Michigan/Wayne State University School of Medicine, Detroit, Michigan; John’s Hopkins Children’s Center, Baltimore, Maryland; Johns Hopkins Hospital, Baltimore, Maryland; Johns Hopkins Medicine, Baltimore, Maryland; Children's Mercy Hospital, Kansas City, Missouri; Children’s Mercy Kansas City, Kansas City, Missouri; Children’s Mercy Hospital, Lees Summit, Missouri; Children's Mercy Hospital, Kansas City, Missouri; Dell Children's Medical Center, Austin, Texas; Children's Mercy Kansas City, Kansas City, Missouri; Ohio State University; Nationwide Children's Hospital, Columbus, Ohio; Children's Mercy Kansas City, Kansas City, Missouri; The Ohio State University College of Medicine; Nationwide Children's Hospital, Columbus, Ohio; Children's Hospital of Michigan, Detroit, Michigan; Children's Hospital of Michigan, Detroit, Michigan; Children's Hospital of Michigan, Flat Rock, Michigan; Department of Surgery, Division of Pediatric Surgery, Children's Hospital of Michigan/Wayne State University School of Medicine, Detroit, Michigan; John’s Hopkins Children’s Center, Baltimore, Maryland; Johns Hopkins Hospital, Baltimore, Maryland; Johns Hopkins Medicine, Baltimore, Maryland

## Abstract

**Introduction:**

It is generally accepted that children with large total body surface area (TBSA) flame burns have worse outcomes as compared to those with scald injury. Anecdotally, we have observed the opposite. We sought to compare outcomes of patients using data from the Pediatric Injury Quality Improvement Collaborative (PIQIC).

**Methods:**

This multicenter, retrospective cohort study collected data from 4 pediatric hospitals participating in PIQIC over a 10 year period. Exclusion criteria were TBSA <15% and mechanism not scald or flame. Patients were categorized by age in years: 0 to <3, toddler; 3 to <10, child; and age ≥ 10. Demographics, clinical features, and adverse events were collected. Models were controlled for percent full thickness burn.

**Results:**

Due to small sample size of age >10, this group was excluded. 164 patients were identified: 96 (59%) with scald and 68 (41%) were flame. Patients in the toddler group were mainly scalds (N=73, 58%) while the child group was mostly flame (N=52, 67%). Only 28% of scald patients had full-thickness burns compared to 67% of flames.

In the scald group, toddlers experienced significantly longer PICU LOS and mechanical ventilator (MV) days than the child group (13 vs. 5 MV days, p < 0.001). Hospital LOS was significantly higher in the child group compared to toddlers. There was trend of higher incidence of sepsis, nosocomial infection, and abdominal catastrophe in toddlers. All mortalities were observed in the child group.

In flame patients, toddlers had no mortalities, cases of sepsis or abdominal catastrophe. Toddlers had significantly shorter hospital LOS (18 vs 25, p< 0.001), PICU LOS (9 [1-14] vs 7 [2-18], p< 0.001), and MV days (6 vs. 9, p< 0.001). Older patients with flame burns had higher %TBSA and full-thickness burns.

Odds of mortality in both mechanisms was significantly increased for higher TBSA. Each 1% increase in TBSA related to an average 3% increase mortality (OR=1.03, 95% CI: (1.00, 1.06), P=0.04). Separating mechanisms, the model determined mortality was affected by age. While scalds had significantly reduced mortality (OR=0.05, 95% CI: (0.00, 0.057), P=0.02), each additional year of age increased mortality by 42% (OR=1.42, 95% CI: (1.07, 1.89), P=0.02). Each additional year of age resulted in an 18% reduction in nosocomial infection (OR=0.83 95% CI: (0.73,0.96), P=0.01).

**Conclusions:**

Given the differences in hospital and PICU LOS, MV days, and complication rates, this study confirms that not all burns carry the same risks across age groups and mechanisms. Despite lower rates of full thickness burns, younger patients with scalds may have a longer PICU course, more time on the ventilator, and increased risk of sepsis, nosocomial infection, and abdominal catastrophe than similarly aged patients with flame burns or older patients with scalds.

**Applicability of Research to Practice:**

Burn mechanism and patient age should be considered in addition to TBSA and burn thickness when caring for a critically ill patient.

